# Decision-Making about the HPV Vaccine among Ethnically Diverse Parents: Implications for Health Communications

**DOI:** 10.1155/2012/401979

**Published:** 2011-11-15

**Authors:** Jennifer D. Allen, Maria de Jesus, Dana Mars, Laura Tom, Lindsay Cloutier, Rachel C. Shelton

**Affiliations:** ^1^Dana-Farber Cancer Institute, 450 Brookline Avenue, Boston, MA 02215, USA; ^2^Harvard Medical School, Boston, MA 02215, USA; ^3^American University, Washington, DC 20016, USA; ^4^Columbia Mailman School of Public Health, New York, NY 10032, USA

## Abstract

*Objective*: To describe parents' knowledge, attitudes, and decision-making with regard to obtaining the HPV vaccine for their daughters. *Methods*: White, Black, and Hispanic parents of daughters who were age eligible to receive the HPV vaccine (9–17 years) were recruited from community settings to participate in focus groups. Parents were asked about knowledge and awareness of HPV, decision-making about HPV vaccine, as well as preferred and actual sources of HPV information. *Results*: Seven focus groups (*n* = 64 participants) were conducted. Groups were segmented by gender (women = 72%) and race/ethnicity (Black = 59%; White = 23%; Hispanic = 19%). Prevalent themes included: insufficient information to make informed decisions; varied preferences for involvement in decision-making; concerns about vaccine safety; mistrust of medical providers and pharmaceutical companies; and mismatch between actual and preferred sources of information. *Discussion*: Improving communication between providers and caregivers and helping parents to access information necessary for informed decision-making, while alleviating concerns about vaccine safety, may help to improve vaccine acceptance.

## 1. Introduction

It is well established that recurrent infection with HPV (human papillomavirus) is responsible for most cervical cancers [1]. With the availability of prophylactic HPV vaccines, there is potential to virtually eliminate disparities in cervical cancer, provided that access is assured and uptake is equitable across population groups.

Currently, routine vaccination is recommended for girls aged 11-12, with “catch up” vaccination recommended between ages 13–26 [2]. Vaccine efficacy is highest when administered prior to sexual début [3]. Given the recommended age for administration, parents have decision-making authority for their minor children. Understanding factors that influence parental decision-making about vaccinating their children may improve HPV vaccination uptake. 

A number of reviews have summarized sociodemographic, cognitive, and attitudinal factors associated with parental acceptance of HPV vaccination [4, 5]. To date, the majority of studies have been conducted among relatively homogeneous samples in clinical settings. We sought to gain a contextualized understanding of vaccine decisions among a racially/ethnically diverse group of parents. We used focus group methodology to illuminate a wide range of perspectives and yield new insights to inform the design of effective interventions and communication strategies [6]. 

## 2. Methods

We developed a focus group guide consisting of open-ended questions (Table 1). Discussion questions were developed from our prior work [7, 8] and existing literature on factors associated with parental acceptance of the HPV vaccine.

During February and May of 2008 we conducted seven focus groups, each with 6–10 parents, Eligible participants were primary caregivers (female or male, hereafter called “parents”) of at least one girl between 9 and 17 years of age. Parents were English or Spanish speaking and self-identified as White, Black, or Hispanic. We recruited participants through health and social service agencies in the Boston metropolitan area through fliers and word of mouth.

Groups were segmented on gender and race/ethnicity, to allow for examination of potential differences across these characteristics. Trained focus group moderators, matched to gender and language of participants, facilitated discussions according to a standardized protocol. Discussions were audio taped, professionally transcribed, and translated. Each discussion lasted 60–90 minutes. Participants received a $50 gift card. The Harvard School of Public Health Institutional Review Board approved this study.

## 3. Analysis

We analyzed focus group transcripts based on procedures outlined by Corbin and Strauss [9]. Several authors (JDA, MDJ, LT) independently examined transcripts to identify themes and create initial coding categories. We subsequently reviewed categories and supporting quotes to develop a refined coding scheme. Through an iterative process, open codes were collapsed into higher-order categories that reflected emergent themes. Where divergent interpretations of themes occurred, we reevaluated and discussed the original transcripts until consensus was achieved. Data collection was stopped at the point of saturation [10].

## 4. Results

### 4.1. Characteristics of Participants

Seven focus groups were conducted with a total of 64 participants. The majority of participants were nonwhite (Black = 59%; Hispanic = 19%; White = 23%) and female (72%). Participants' mean age was 46. Nearly all (98%) reported having health insurance (data not shown).

## 5. Themes

Themes are presented in the order of discussion, which was driven by the focus group guide. We note where themes were salient or emerged exclusively in particular groups. Refer to Table 2 for sample quotes supporting themes.

### 5.1. Insufficient Information to Make Informed Decisions

The majority of parents felt that they did not have adequate information about HPV or the vaccine to make an informed decision about vaccinating their children. A number of participants confused HPV with HIV or Hepatitis. Many of the men had never heard of HPV prior to the focus group. In each of the discussions among women, at least one person shared that they had allowed one or more of their daughters get the vaccine, although they felt that they had been inadequately informed or were unprepared for the decision. In some cases, participants reported that they were not given the opportunity to be involved in decision-making. In other instances, parents reported that they were given a fact sheet, but not the opportunity to ask questions.

### 5.2. Responsibility for Decision-Making

Parents expressed a wide range of perspectives in terms of the extent of involvement inthe decision-making process they wanted for themselves and their daughters. In general, fathers indicated that they would defer decision-making to female family members (e.g., grandmothers, godmothers) or trusted friends, particularly among the African American and Hispanic groups. The desire for a collaborative decision-making process between the provider, parent, and daughter was commonly expressed by Hispanic mothers. For example, several Hispanic mothers related stories about concern when health care providers discussed the vaccine with their daughter privately and not involving the parent in discussions. This was a cause of concern for these parents, who felt that providers should have involved both the parent and daughter in the decision. The opinion that the primary female caregiver should make decisions (without necessarily involving the daughter) appeared to be most common among African American women. A view more commonly expressed among White women was that the decision could or should be left to the daughter, depending on age and maturity level, and as long as she was adequately informed about the vaccine.

### 5.3. Concerns about Vaccine Safety

All of the groups raised concerns about vaccine safety. Many shared the opinion that there may be vaccine side effects yet unknown. Concerns about unknown, long-term side effects were most frequently discussed among African American groups. The most prominent vaccine safety concerns among Hispanic mothers were birth defects and future reproductive health.

### 5.4. Mistrust of Medical Providers and Pharmaceutical Companies

Mistrust of medical providers was expressed by at least one individual in each of the groups. Females, in particular, expressed skepticism and surprise that they had not previously heard about HPV until the vaccine became available. Some expressed anger at providers for not having previously discussed this “killer virus.” Participants also discussed their mistrust of pharmaceutical companies, with some perceiving distribution of vaccine as solely profit driven. Pharmaceutical company mistrust appeared most prevalent among African American groups, where participants drew parallels between being injected with a virus and the Tuskegee experiment.

### 5.5. Mismatch between Actual Versus Preferred Sources of Information

The majority of parents reported that they preferred to receive vaccine information directly from their daughter's health care provider. At the same time, there was much discussion about feeling rushed at medical appointments, not having sufficient opportunities to ask questions, and as previously mentioned, a sense that health care providers had been withholding information about HPV. While providers were the preferred sources of information, the vast majority of participants stated that their primary source of information about HPV and the vaccine had been television and radio advertisements. Participants in all groups reported seeing advertisements for the vaccine and specifically identified the “One Less” campaign. Many expressed concern that advertisements provided insufficient, misleading, or inaccurate information.

## 6. Discussion

In this qualitative study of ethnically diverse parents, knowledge and awareness of HPV and the vaccine was low. Parents almost universally reported feeling that they did not have sufficient information to make an informed decision about vaccinating their daughter(s). Concerns about vaccine safety and potential side effects were also prevalent. Often, these concerns were discussed in the context of mistrusting pharmaceutical companies and a concern that the vaccine is being promoted solely for profit. In these respects, our findings are consistent with recent studies of parental knowledge and attitudes toward the HPV vaccine [5, 11]. 

Our findings shed new light on parental desire for, and experiences with participation in decision-making. Although this study was not specifically designed to test differences in opinion across gender or racial/ethnic groups, we noted variations in themes between groups.

Hispanic mothers more often reported minimal involvement in decision-making despite a desire to take an active role. Most Hispanic and African American men preferred to defer decisions to female caretakers. Whereas Hispanic mothers reported more concern over birth defects and reproductive health. Parents in all groups reported learning about HPV from media sources, yet almost all reported a preference for receiving information from their daughter's health care provider. 

Prior to discussion of potential implications, limitations of this study must be noted. This was a sample of convenience, which limits the generalizability of our results. Our goal, however, was not to obtain a representative sample, but rather to hear from parents from a variety of backgrounds and who had diverse perspectives and experiences. It is possible that study volunteers were more interested in HPV and more knowledgeable about the topic compared to the general parent population. If, indeed, participants are assumed to be more knowledgeable than the general population, these findings further emphasize the need for HPV vaccine interventions, as levels of knowledge and awareness were low. Lastly, we were not able to explore inter-ethnic differences across racial groups (e.g., by country of origin or region). We recognize the heterogeneity within each of the groups but were limited in our analysis by sample size. Nevertheless, our data are useful in generating hypotheses that can be tested in future research and for informing health communication intervention development [6]. 

There are several important implications of this research. Parents need additional information about the vaccine, its benefits, and limitations. Parental reports of inadequate information or involvement in decision-making is cause for concern for many reasons. First, participation of parents in vaccine decisions could increase providers' abilities to assess risks of side effects. For example, providers need to be alerted to a child's allergies or any major illnesses, which could affect vaccine response [2]. Second, parents need to be informed of the potentially serious (but rare) allergic reactions that can occur, as well as the limitations of the vaccine (i.e., it does not protect against other sexually transmitted infections). Third, increased parental knowledge about the vaccine could result in improved rates of vaccine series completion. Currently, most who initiate the vaccine's series fail to receive the required three-dose regimen [12]. 

Parents reported a strong preference to receive HPV-related information directly from their daughters' health care provider. As such, interventions aimed at improving provider communications with parents about HPV are needed. Provider endorsement has been found to be an important driver of HPV vaccination in previous studies [5]. Providers need information and skills to effectively disseminate accurate information, help manage parental uncertainty, respond to emotional concerns, and facilitate informed decision-making among parents. However, given the mistrust expressed among some groups toward providers, interventions will also need to foster strong provider-patient relationships. While time constraints are a potential barrier to developing relationships with parents, there are ways to address this. For example, provider vaccine recommendations can be followed by reminder mailings and parent education on how to best prepare for a clinical encounter.

Our finding that female caregivers often hold primary responsibility for health decisions, particularly in relation to the sexual health of their daughters, highlights the importance of targeting HPV health communications. For example, the importance of vaccinating young girls could be discussed with mothers when they themselves present for cervical cancer screening. In addition, the finding that many parents rely on female family members and friends for input on health-related decisions suggests that targeting key members within an individual's social network could be potentially effective for the dissemination of HPV vaccine information and adherence to provider recommendations. 

Finally, to address parental mistrust of pharmaceutical companies, it will be important to deliver interventions that are not associated with or financially tied to the pharmaceutical industry. This may help to improve program credibility and mitigate mistrust. Given some of the public's mistrust of vaccines in general, providing information through trusted and credible sources will be critical. 

In conclusion, given that there is variation among ethnically diverse parents in the degree of desired involvement in decision-making, it would be prudent for providers to inquire about and respect preferences for involvement. Understanding the diverse models of parental decision-making about HPV vaccination would enable the development and evaluation of much needed targeted health communication interventions to promote vaccine uptake and completion.

## Figures and Tables

**Table 1:** Focus group questions.

Construct	Sample question
Sources of health information	Where do you get information about your daughter's health? What is the most credible/believable source of information?

Decision-making about daughter's health	Who makes decisions about your daughter's health? Under what circumstances would you involve your daughter in making a decision about her health care?

Awareness and knowledge of HPV	What do you know about HPV or human papillomavirus? How is it spread? Who is vulnerable to it? What health problems can it cause?

Awareness and knowledge of HPV vaccine	Have you heard about a vaccine for HPV? What have you heard? Who is this vaccine for? What does it do?

Social and subjective norms about HPV vaccination	Are people in your community getting their daughters vaccinated? Why or why not? What would your (spouse/partner; family members; friends) think about your getting your daughter vaccinated for HPV?

Barriers to vaccination	Is it difficult to get this vaccine? Why or why not?

Desired information	What would you need to know before making a decision about vaccinating your daughter(s)?

Preferred sources and settings for HPV information delivery	What would be the best way to deliver information about the HPV vaccine to parents? Where do you prefer to receive information? From what source?

**Table 2:** Focus group themes.

Theme	Example quotes
Insufficient information to make informed decisions	“I didn't give it [HPV vaccine] to my daughter when the doctor asked because I wasn't aware of the consequences. I need more research on it before I put her through something like that.” *(Black female) * “Men need to get information so that we can get more educated about HPV.” *(Black male) * “She got [the vaccine], but I am not well-informed. I don't know what effects it could have on her. I was not given the information …” *(Latino female) * “My daughter just got the shot. And I don't even know what she got … You know I don't even know how to say it. Papee-glorenoma virus” *(White female) * “They don't explain much to you. They give you a paper with the risks …” *(White female) *

Involvement in decision making	“I leave it up to my daughter and her mother to decide what to do about the HPV vaccine.” *(Black male) * “I make that decision. Her body is mine until she's 18 years old.” *(Black female) * “I would ask some of the women in my family for their opinion … my aunt. I would get her opinion and my sister's, too.” *(Latino male) * “When I went to the doctor with my daughter, I was asked to leave the office as the doctor wanted to have a private conversation about the HPV vaccine with her. When they were finished, I came back in. I asked the doctor, “What's going on?” The doctor didn't tell me anything … I am kind of confused … because the doctor didn't say anything to me about it [vaccine].” *(Latino female) * "I think it's better between the daughter and the health care provider” *(White male) * “I would let the decision be up to her, but she should be informed. The more informed she is, the better off she'll be making the decision, and she won't feel forced by you, or anybody else …” *(White female) *

Concerns about vaccine safety	“My first thought was, I am sendin' my ten-year old to this clinic to put dead HPV cells in her. What if the HPV that they are shooting in her body … what if it comes to life?” *(Black female) * “[They] tried to give me an HPV shot, along with others, while in prison. It was a trial.” *(Black male) *

Mistrust-pharmaceutical companies	“… all those pharmaceuticals care about, let's be honest, is money.” *(Black female) * “Are the companies who are putting the HPV vaccine out here … are they doing it just for a profit? Or do they actually really care about treatment and prevention?” *(Black female) * “If it's been around and studied for this long, then why is it just now starting to hit the market, and why is it just now starting to get pushed?” *(Black male) *

Mistrust-medical providers	“I was pregnant thirteen times but … it's [HPV] never, ever once [taps on table for emphasis] come up with my doctor! Never once have they even mentioned [HPV]!” *(Black female) *

Sources of information	“I don't think the TV commercials explain enough, all they say is “one less, one less, one less.” I would like to know how long the vaccine is good for and its effectiveness.” *(Black female) * “I know about HPV and the vaccine because they advertise the HPV vaccine a thousand times on Univision and Telemundo.” *(Latino female) * “Knowledge is power. We should be getting information about the HPV vaccine from our girls' doctors.” *(White female) *

Desired educational materials and strategies	“You can't use big words, you need to make it understandable so that people can understand [the vaccine information] and make decisions.” *(Black female) * “For me, the more information channels there is, the better: written, video, discussions…Sometimes you don't understand something one way and you understand it in a different format.” *(Latino male) * “Sometimes, because you don't know the language, you don't even ask.” *(Latino female) *
